# JDS Guyane 2021 - 4^e^ journée des travaux scientifiques des soignants de Guyane - Cayenne - 25 Juin 2021

**DOI:** 10.48327/mtsibulletin.2021.121

**Published:** 2021-08-12

**Authors:** A. Lucarelli, A. Fremery, A. Argoubi, S. Bernard, C. Bertin, M. Boutrou, P. Cousin, M. Douine, L. Hureau Mutricy, R. Mutricy, C. Obert-Marby, L. Osei, M.-H. Poirot-Fouillet, T. Bonifay, L. Epelboin

**Affiliations:** 1 Consultations adultes spécialisées et COREVIH Guyane, CH de Cayenne, Guyane; 2 Service d'accueil des Urgences, CH de Cayenne, Guyane; 3 Mairie de Cayenne, Guyane; 4 Réseau Périnat, Cayenne, Guyane; 5 Unité des maladies infectieuses et tropicales, CH de Cayenne et Service de Médecine, Centre hospitalier de l'Ouest Guyanais, Saint Laurent du Maroni, Guyane; 6 Unité des maladies infectieuses et tropicales, CH de Cayenne et Service de Médecine, Centre hospitalier de Kourou, Kourou, Guyane; 7 Centres délocalisés de prévention et de soins, CH de Cayenne, Guyane; 8 Centre d'investigation clinique Antilles Guyane Inserm 1424, CH de Cayenne, Guyane; 9 COREVIH Guyane, CH de Cayenne, Guyane; 10 Service d'accueil des Urgences, CH de Cayenne, Guyane; 11 Permanences d'accès aux soins de santé, Centre hospitalier de l'Ouest Guyanais, Saint Laurent du Maroni, Guyane et Département universitaire de médecine générale, Université des Antilles, Pointe-à-Pitre, Guadeloupe; 12 Service de pédiatrie et Centre d'investigation clinique Antilles Guyane Inserm 1424, CH de Cayenne, Guyane; 13 Unité des maladies infectieuses et tropicales, CH de Cayenne, Guyane; 14 Unité sanitaire en milieu pénitentiaire, Centre pénitentiaire de Guyane et Centre d'investigation clinique Antilles Guyane Inserm 1424, CH de Cayenne, Guyane; 15 Unité des maladies infectieuses et tropicales, Centre d'investigation clinique Antilles Guyane Inserm 1424, Centres délocalisés de prévention et de soins, CH de Cayenne, Guyane

Créée en 2017 par une petite équipe de médecins de Guyane, la Journée des travaux scientifiques des jeunes médecins de Guyane (Nos internes ont du talent) a pris un nouvel essor en 2021 en devenant la 4^e^ journée des travaux des soignants de Guyane ou JDS Guyane (Fig. 1, 2, 3, 4). En effet la session 2021 a été novatrice à plusieurs égards. Tout d'abord, la journée s'est ouverte à tous les corps de métier dans le domaine de la santé, ainsi des pharmaciens, infirmiers et sages-femmes ont, pour la première fois, contribué à la réalisation et à la réussite de cette journée. Malgré une succession d'annulation et de report du congrès 2020, la journée a été maintenue en 2021 et organisée à la fois en présentiel et en distanciel, permettant la participation de plus de 350 personnes. Cet événement, sans précédent, a permis de proposer le superlatif de 1^er^ e-congrès guyanais.

**Figure 1:**
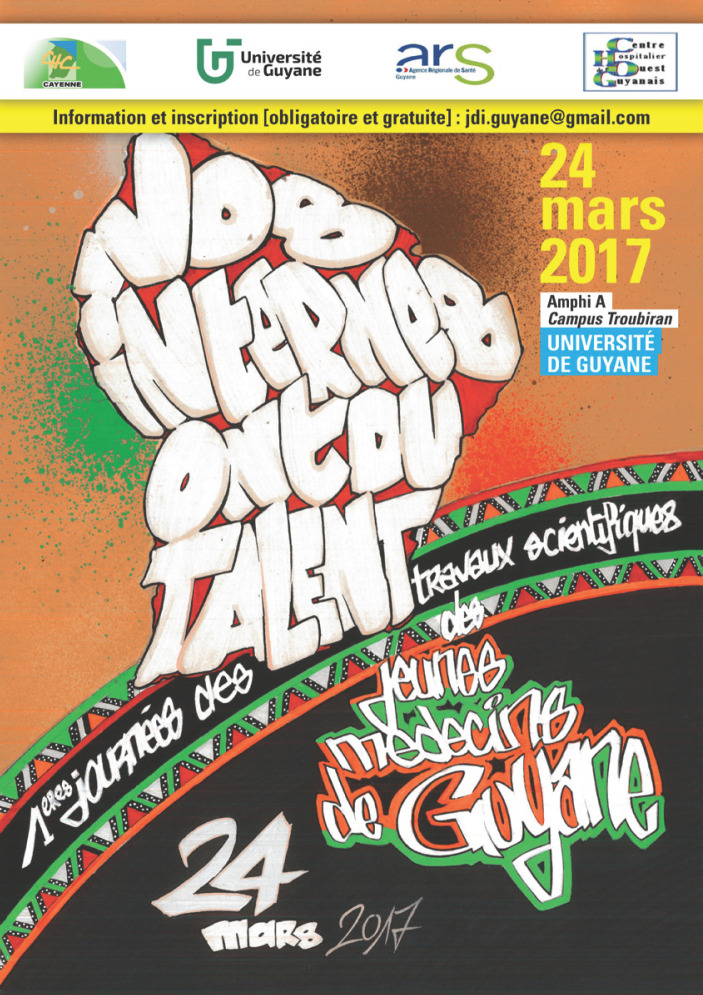
Affiche de la 1^re^ Journée des travaux scientifiques des jeunes médecins de Guyane: Nos internes ont du talent (24 mars 2017) Poster of the 1^st^ Day dedicated to the Scientific Works of Young Doctors in French Guiana: Our Residents’ Got Talent (March 24, 2017)

**Figure 2:**
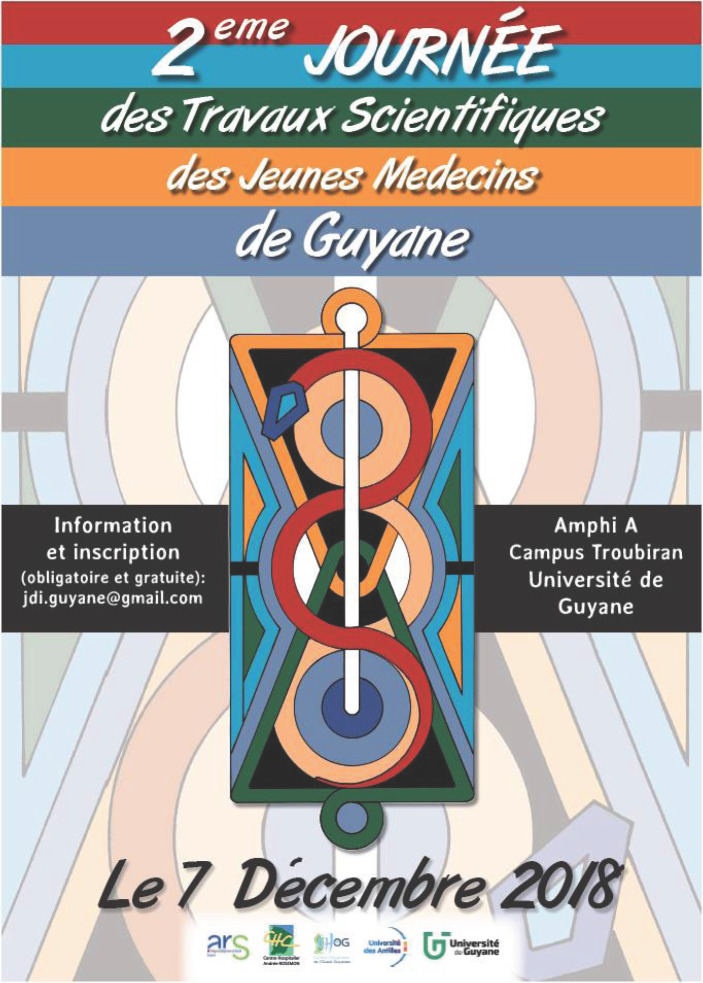
Affiche de la 2^e^ Journée des travaux scientifiques des jeunes médecins de Guyane (7 décembre 2018) Poster of the 2^nd^ Day dedicated to the Scientific Works of Young Doctors in French Guiana: (December 7, 2018)

**Figure 3:**
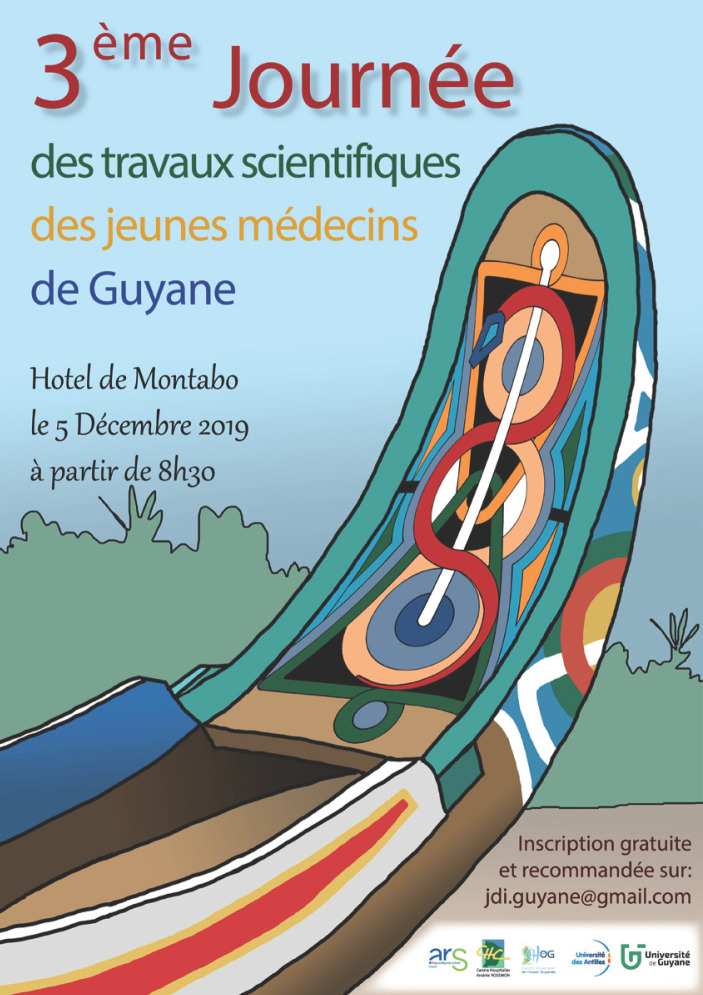
Affiche de la 3^e^ journée des travaux scientifiques des jeunes médecins de Guyane (5 décembre 2019) Poster of the 3^rd^ Day dedicated to the Scientific Works of Young Doctors in French Guiana: (December 5, 2019)

**Figure 4:**
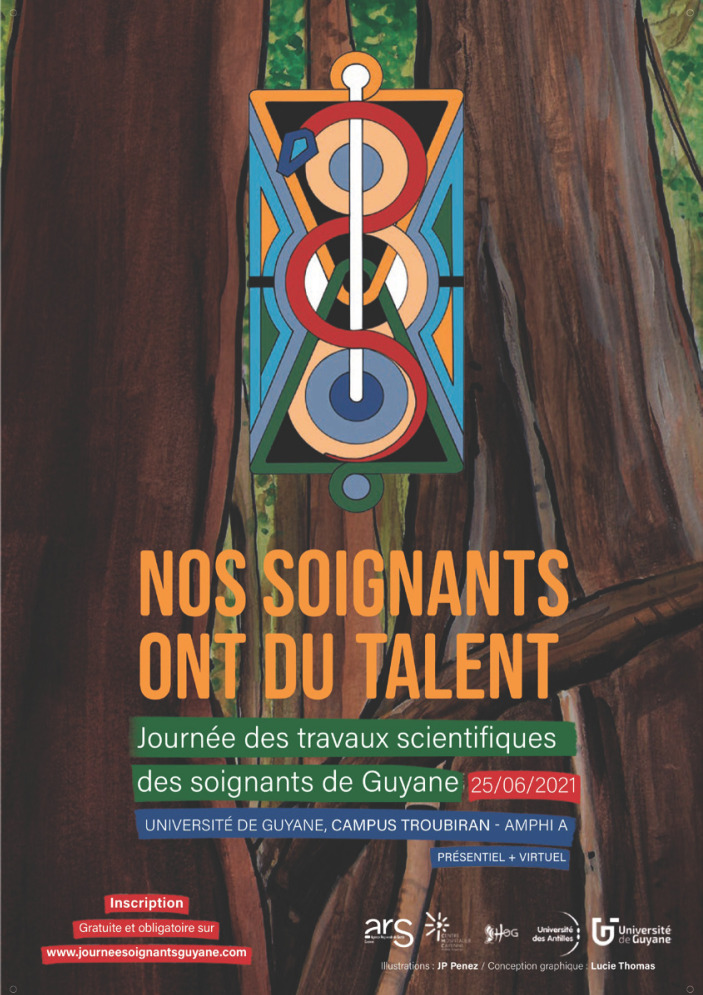
Affiche de la 4^e^ Journée des travaux scientifiques des soignants de Guyane: Nos soignants ont du talent (25 juin 2021) Poster of the 4^th^ Day dedicated to the Scientific Works of Caregivers in French Guiana: Our Caregivers’ Got Talent (June 25, 2021)

Le cru 2021 n'a pas eu à rougir de la qualité et de la diversité des travaux présentés; le choix entre communication orale et sous forme de poster étant toujours aussi difficile pour le comité scientifique.

Cette année, la journée a été répartie en quatre sessions. Une première partie dite « intoxication » a abordé des problématiques de santé publique importantes: les intoxications accidentelles aux plantes, l’épidémiologie des mules ou body-packers de cocaïne et l'exposition au plomb chez les enfants. La session « infectiologie » a abordé des thèmes à la fois tropicaux: investigation d'une épidémie de leishmaniose cutanée chez des militaires, utilisation du β-D-glycane dans le diagnostic de l'histoplasmose du sujet VIH; mais aussi l’étude de pathologies cosmopolites en contexte tropical: complications infectieuses chez les transplantés rénaux vivant en région amazonienne et épidémiologie de la maladie de Kaposi. Une session dite « médecine ambulatoire » a ensuite abordé la thématique des AVC chez les patients porteurs d'un syndrome drépanocytaire majeur, celle du suivi médical des enfants en communes isolées et celui du ressenti des médecins prenant en charge des urgences vitales dans des communes isolées. La dernière session, dédiée aux IST, a été le théâtre de trois présentations, l'une sur la comparaison des profils des personnes vivant avec le VIH suivi dans le CH de Saint-Laurent-du-Maroni et celui de Cayenne, une étude sur les facteurs associés à la non-récupération des résultats de dépistage des IST dans l'Ouest Guyanais et enfin une présentation d'une étude sur l’épidémiologie des infections génitales à chlamydia et gonocoque à Maripasoula.

Si le retour, tant oral que sur les réseaux concernant ces Journées des travaux scientifiques des soignants de Guyane est toujours positif, le retour sur les publications dans des revues internationales indexées ne dément pas cette impression. Ainsi 84 % (16/19) des communications de la saison 2017 ont été publiées et sont disponibles sur Pubmed, 32 % (9/28) pour la saison 2018, 20 % (5/25) pour la saison 2019 et déjà 19 % (4/21) pour la saison 2021, et ce, sans compter sur un certain nombre de communications des années précédentes en cours de soumission ou de relecture.

Ainsi le succès et la qualité des JDS Guyane continuent à mobiliser des invités du monde entier: le distanciel a permis un auditoire de tous horizons, se connectant depuis la Guyane, les Antilles, la France hexagonale, mais aussi depuis le Congo, la Côte d'Ivoire et la Thaïlande ! En espérant que la situation sanitaire permettra une saison 2022 aussi réussie que celle de cette année, avec comme objectif de promouvoir encore plus les travaux des non-médecins: sages-femmes et infirmiers notamment. *Nou ké tchimbé red, pa ké moli,* comme on dit couramment en Guyane: on tiendra bon, on ne faiblira pas pour la prochaine saison 2022 !

## Remerciements

Les organisateurs remercient le comité scientifique pour la relecture des résumés: Antoine Adenis, Camille Agostini, Olivier Angenieux, Anna Auguste, Stéphanie Bernard, Vincent Berot, Claire Boceno, Timothée Bonifay, Mathilde Boutrou, Gabriel Carles, Bertrand De Toffol, Narcisse Elenga, Loïc Epelboin, Alexis Fremery, Michele Goldzack, Véronique Lambert, Aude Lucarelli, Elise Martin, Christian Marty, Caroline Mislin-Trish, Rémy Mutricy, Alice Sanna et Camille Thorey.

Les organisateurs remercient les modérateurs pour l'animation des sessions: Jérémie Bouche, Adriana Gonzalez, Hatem Kallel, Richard Naldjinan, Lindsay Osei, Frédérique Perotti et Camille Thorey ainsi que Antoine Adenis pour avoir été le grand témoin de ces journées.

Les organisateurs remercient M. Christophe Robert (directeur général du Centre hospitalier de Cayenne), M. Antoine Primerose (président de l'Université de Guyane) et Mme Clara De Bort (directrice générale de l'Agence régionale de santé Guyane), pour l'introduction enjouée et enthousiasmée de cette journée.

Les organisateurs remercient l'Agence régionale de santé Guyane pour son soutien indéfectible et son mécénat sans faille à ces journées, l'Université de la Guyane pour les locaux et l'association Carbu pour la gestion financière.

Les organisateurs remercient M. Jean-Pierre Penez pour la réalisation de l'affiche et la boite Aéroprod qui a permis la tenue en ligne de cette journée.

Les organisateurs tiennent enfin à remercier tout particulièrement Mme Bénédicte Sauvage, de la boite Bcom, pour son engagement sans faille à nos côtés, dans tous les aspects logistiques, numériques et graphiques de la réalisation de ces journées et de la création du nouveau site dédié à ces journées: https://www.journeesoignantsguyane.com.

