# Supplementary material for: JDS Guyane 2021 - 4th day dedicated to the scientific works of caregivers in French Guiana - Cayenne - June 25, 2021
Source: Med Trop Sante Int. 2021 Aug 12;1(3):mtsibulletin.2021.121. [Article in French] doi: 10.48327/mtsibulletin.2021.121 (PMC9128485; doi:10.48327/mtsibulletin.2021.121)
Supplement: Related texts

# Research article: JDI-05: Analyse du ressenti de témoins non professionnels de santé, ayant reçu des consignes de réalisation de manœuvre de réanimation sur un patient en ACR, en extra-hospitalier

**Published:** 2021-08-12

**Authors:** S. Belle, P. Chesneau

**Affiliations:** Service d'accueil des urgences SAMU, Centre Hospitalier de Cayenne, Guyane française, France

## Introduction

L'objectif est de décrire le ressenti des personnes face à un ACR et d’évaluer le stress post-traumatique induit par cette expérience.

## Matériel et Méthode

Une méthodologie qualitative par saturation a été choisie. Sont inclus les non-professionnels de santé ayant reçu des consignes de réaliser un massage cardiaque par le 15.

## Résultats

Dix entretiens ont été réalisés. Six témoins ont massé et 4 ont reçu les consignes, mais n'ont pas massé. Les 6 personnes ayant massé avaient reçu une formation. Les relations des témoins avec les victimes étaient le plus souvent familiales. Les émotions ressenties en fonction du moment ont été étudiées et présentées. L’évaluation du score PCL-S a permis d'identifier trois syndromes de stress post traumatiques (SPT).

## Discussion

La reconnaissance de l'ACR, et la réalisation du MCE entraînent des séquences émotionnelles qui se modifient au cours du temps. Nos résultats concordent avec l’étude de Mathiesen (2016) concernant le ressenti des témoins et la variation de ressenti en fonction du niveau de compétence perçue après la réanimation. Les témoins n'ont pas assisté à la réanimation spécialisée. Il n'y a pas eu de proposition de débriefing ni de prise en charge de SPT.

## Conclusion

L'analyse de la dynamique des émotions met en évidence la difficulté émotionnelle ressentie lors de la prise en charge d'un ACR par des témoins non professionnels. Des séquelles psychologiques sont constatées chez les témoins interrogés.

# Research article: JDI-11: Etude rétrospective des cas d'intoxications aux plantes en Guyane et mise à disposition d'outils pratiques à destination des hôpitaux

**Published:** 2021-08-12

**Authors:** T. Blaise, G. Odonne, P. Champy, B. Weniger, M.-A. Tareau, M. Omri, H. Kallel, E. Houel, J. Langrand, M. Douine

**Affiliations:** 1 Centre d'investigation clinique Antilles Guyane, CIC INSERM 1424, Centre hospitalier Andrée Rosemon, Cayenne, Guyane française, France; 2 LEEISA (Laboratoire écologie, évolution, interactions des systèmes amazoniens), CNRS, Université de Guyane, IFREMER, Cayenne, Guyane française, France; 3 Université Paris-Saclay, CNRS, BioCIS, 92290 Châtenay-Malabry, France; 4 Faculté de Pharmacie de Strasbourg, UMR 7200 - Laboratoire d'innovation thérapeutique BP 60024F – 67401 Illkirch cedex, France; 5 Emergency Department, Centre hospitalier de Cayenne, Cayenne 97300, Guyane française, France; 6 Intensive Care Unit, Cayenne General Hospital, Cayenne, Guyane française, France; 7 Poison Control Center of Paris, Federation of Toxicology, Fernand-Widal-Lariboisière Hospital, AP-HP, INSERM UMRS-1144, University of Paris, Paris, France

## Introduction

En Guyane française, l'utilisation de plantes médicinales est largement répandue. Malgré une bonne connaissance de la pharmacopée traditionnelle par les guyanais, les consultations aux urgences pour des intoxications liées à des plantes sont fréquentes. La prise en charge des patients est alors principalement symptomatique, et l'absence de guidelines ne facilite pas l'instauration d'un traitement adapté.

## Méthodologie

Étude rétrospective des cas d'intoxications incriminant des plantes en Guyane française depuis 2000. Interrogation de plusieurs bases de données (BDD) à partir de mots-clefs et de codes CIM10. Constitution d'une BDD commune. Pour chaque cas, calcul d'un score de gravité. Pour chaque plante incriminée, calcul de fréquence et de gravité moyenne.

## Résultats

À partir des 4 bases de données interrogées, 173 cas d'intoxications ont été retenus dont 100 cas avec une identification de l'espèce botanique. Une liste de 12 plantes impliquées dans des intoxications graves et/ou fréquentes a été constituée. Afin de faciliter l'identification botanique des plantes incriminées dans les intoxications, un système de mise en relation soignants, centres antipoison et botanistes a été développé. Cette « Phytoliste » permet d'obtenir rapidement une identification botanique formelle à partir d'une photo de plante. Parallèlement, des fiches pratiques ont été rédigées. Elles synthétisent pour chaque plante retenue les caractéristiques toxicologiques et botaniques ainsi que la symptomatologie et la prise en charge de ces intoxications.

## Discussion et Conclusion

Afin de proposer un outil pratique d'aide à la prise en charge des cas d'intoxications aux plantes, un classeur contenant les fiches pratiques des 12 plantes identifiées sera mis à disposition du service des urgences du CHC. Ce classeur contiendra également les modalités d'utilisation de la Phytoliste. Des séances de formation accompagneront la mise à disposition du classeur. À terme, ces outils seront également proposés aux CDPS ainsi qu'au CHK et au CHOG.

# Research article: JDI-17: Évaluation qualitative du ressenti des médecins concernant la prise en charge des urgences vitales dans les CDPS de Guyane Française

**Published:** 2021-08-12

**Authors:** R. Blanc, A. Fremery, J.-M. Pujo

**Affiliations:** Service d'accueil des urgences, SAMU-SMUR 973, Centre hospitalier de Cayenne, Cayenne, Guyane française, France

## Introduction

La population médicale en Centre délocalisé de prévention et de soins (CDPS) est principalement composée de médecins généralistes jeunes et peu formés à la médecine d'urgence. Les urgences vitales sont fréquentes dans nos centres de santé et ces médecins sont amenés à prendre à charge des patients graves jusqu’à l'arrivée du SMUR pendant des périodes parfois prolongées. L'objectif de notre étude est de décrire les situations d'urgences vitales vécues en CDPS et d'identifier des pistes d'améliorations de prise en charge de ces patients.

## Matériel et Méthode

Nous avons réalisé une étude descriptive au moyen d'un questionnaire informatique avec 21 questions dont 2 ouvertes. Il a été diffusé à l'aide d'une mailing list de 310 contacts de médecins ayant travaillés dans les CDPS de 2010 à 2019. Nous avons réalisé une analyse quantitative, qualitative et empirique.

## Résultats

Nous avons obtenu 90 réponses et analysé 87. Le taux de participation était de 28 %. La majorité des médecins étaient des généralistes (72 %) ayant moins de 40 ans (69 %) sans formation de médecine d'urgence (76 %) et ayant déjà travaillé en CDPS pour 56 % d'entre eux. Les urgences vitales majoritairement rencontrées étaient des comas, des polytraumatisés, des urgences gynécolologiques, des chocs septiques et hémorragiques. La majorité (67 %) des médecins ont rapporté avoir été inconfortables durant la prise en charge dont 1/3 des médecins urgentistes. Parmi eux, 40 % ont été confrontés à l'intubation orotrachéale et seuls 29 % des médecins non urgentistes ont réussi. La relation avec le SAMU a été jugée très majoritairement adaptée (93 %). Plus d'un médecin sur 5 ne renouvellera pas son contrat en CDPS du fait du vécu des urgences vitales. Afin d'améliorer la prise en charge, les médecins sont favorables à la présence de fiches « REFLEXES », à une formation en pré-affectation à l'IOT et au KTIO, à de courtes formations aux déchoquages, à des alternatives à l'IOT tel que le Combitub et à une relation privilégiée par de la télémédecine à l'aide de caméra.

## Conclusion

Les urgences vitales sont des situations stressantes pour les médecins. L'amélioration de leurs prises en charge passe par la mise en place de mesures avant pendant et après l'affectation des médecins en CDPS. La majorité de ces mesures sont en places actuellement ou sont en projet et en cours d’évaluation.

# Research article: JDI-01: Complications infectieuses chez les transplantés rénaux en Guyane Française: quelles particularités par rapport à la métropole?

**Published:** 2021-08-12

**Authors:** L. Cachera, A. Adenis, J.-M. Dueymes, D. Rochemont, B. Guarmit, R. Roura, M. Meddeb, M. Nacher, F. Djossou, L. Epelboin

**Affiliations:** 1 Université Paris Descartes, Sorbonne Paris Cité, Paris, France; 2 Service de maladies infectieuses et tropicales, Centre hospitalier Andrée Rosemon, Cayenne, Guyane française, France; 3 Centre d'investigation Clinique « Inserm CIC 1424 », Cayenne, Guyane française, France; 4 Service de Néphrologie, Centre hospitalier Andrée Rosemon, Cayenne, Guyane française, France; 5 Coordination Régionale de lutte contre le Virus de l'immunodéficience humaine, « COREVIH », Centre hospitalier Andrée Rosemon, Cayenne, Guyane française, France; 6 Association traitement insuffisance rénale Guyane « A.T.I.R.G », Cayenne, Guyane française, France; 7 Clinique Véronique, Cayenne, Guyane française, France; 8 EA3593, Epidémiologie des parasitoses et des mycoses tropicales, Faculté de médecine des Antilles et de la Guyane, Cayenne, Guyane française, France

## Introduction

Les complications infectieuses en transplantation rénale n'ont jamais été étudiées sur le plateau des Guyanes. L'objectif principal de l’étude était de décrire le spectre étiologique des infections ayant nécessité une hospitalisation chez les patients transplantés rénaux en région tropicale amazonienne.

## Matériel et Méthode

Tous les patients porteurs d'un transplant rénal fonctionnel entre 2007 et 2019 et résidant en Guyane française ont été inclus rétrospectivement. Les hospitalisations pour un motif infectieux ont été recueillies dans les 4 principaux centres médicaux de Guyane, après un délai de 3 mois suivant chaque transplantation pour ne pas recueillir les infections acquises sur le site du centre greffeur (Métropole ou Guadeloupe). Les infections associées aux soins n'ont pas été analysées.

## Résultats

Quatre-vingt-deux patients, transplantés entre 1987 et 2018, ont été inclus. Quatre-vingt-sept infections ont été recueillies chez 42 patients (51,2 %), correspondant à un taux d'incidence de 0,18 infection par patient-année et à une moyenne de 2,07 infections par patients infectés. Le délai moyen entre la transplantation et l'infection était de 8,5 ans. Le site initial de l'infection était urinaire, dans 28,7 % des cas (25/87), pulmonaire dans 21,8 % des cas (19/87) et cutanéomuqueux dans 13,8 % des cas (12/87). Quand elles étaient documentées (48/87), les infections bactériennes étaient majoritaires (35/48, 72,9 %) suivies des infections virales (8/48,16,7 %), fongiques (4/48, 8,3 %) et parasitaires (1/48, 2,1 %). Les infections endémiques dites « tropicales » correspondaient à 5,7 % des infections (5/87). Les entérobactéries étaient les bactéries les plus fréquentes (22/35, 62,9 %). Les infections à VZV étaient la première cause d'infections virales (4/8). *Histoplasma capsulatum* était le champignon le plus souvent isolé (2/4). La mortalité intra-hospitalière était de 6,9 % (6/87).

## Conclusion

Cette étude suggère que le spectre des infections bactériennes et virales chez les patients transplantés en Guyane diffère peu de celui de la métropole, avec une prédominance des infections urinaires et pulmonaires. Cependant, près de 6 % des infections étaient dues à des infections endémiques tropicales, et les infections fongiques se démarquaient par une part prépondérante d'H. *capsulatum.* D'avantages d’études sur le risque infectieux fongique des transplantés rénaux en Amazonie sont nécessaires pour préciser le poids des mycoses endémiques chez ces patients fragiles.

# Research article: JDI-03: Présentation simultanée d'une infection à Parvovirus B19 et d'un lupus érythémateux systémique chez un patient: description et revue de la littérature

**Published:** 2021-08-12

**Authors:** P. Chabert, H. Kallel

**Affiliations:** Intensive Care Unit, Cayenne General Hospital, Cayenne, Guyane française, France

## Introduction

Nous présentons le cas d'une femme de 25 ans sans antécédents médicaux, présentant une insuffisance respiratoire aiguë nécessitant une ventilation mécanique. Le dépistage étiologique a révélé une primo-infection à parvovirus B19 (PVB19) et une poussée concomitante de lupus érythémateux disséminé (LED). Nous discutons des interactions causales entre le PVB19 et le LED dans la pathogenèse de la maladie. La difficulté de diagnostiquer une poussée inaugurale de LED concomitante à une infection par le PVB19 peut entraîner un retard de diagnostic et de traitement. Inversement, le surtraitement d'une infection au PVB19 imitant le LED par des agents immunosuppresseurs peut être très préjudiciable.

# Research article: JDI-15: Infections de la peau et des tissus mous associées à *Aeromonas* en Guyane Française: une étude rétrospective sur 11 ans

**Published:** 2021-08-12

**Authors:** M. Devos, V. Sainte-Rose, H. Kallel, C. Mayence, H. Ouedraogo, F. Djossou, M. Demar, P. Couppié, R. Blaizot

**Affiliations:** 1 Service de dermatologie, Hôpital Andrée Rosemon, Cayenne, Guyane française, France; 2 Laboratoire de bactériologie, Hôpital Andrée Rosemon, Cayenne, Guyane française, France; 3 Service de réanimation, Hôpital Andrée Rosemon, Cayenne, Guyane française, France; 4 Service d'orthopédie, Hôpital Andrée Rosemon, Cayenne, Guyane française, France; 5 Service de maladies infectieuses, Hôpital Andrée Rosemon, Cayenne, Guyane française, France; 6 Equipe EA 3593, Ecosystèmes Amazoniens et Pathologies Tropicales, Université de Guyane française, Cayenne, Guyane française, France

## Introduction

Les bactéries du genre *Aeromonas,* largement répandues dans l'eau et le sol, ont été associées à des infections de la peau ou des tissus mous. Toutefois, il existe peu de données sur ces infections, en particulier en Amérique du Sud. Nous avons étudié les caractéristiques épidémiologiques, cliniques, les facteurs de risque d’évolution défavorable et la sensibilité aux antibiotiques des *Aeromonas* impliqués dans les infections de la peau et des tissus mous en Guyane.

## Méthodologie

Cette étude rétrospective a été menée entre décembre 2007 et juin 2018 en Guyane. Les patients inclus avaient une infection de la peau ou des tissus mous avec au moins une culture positive à *Aeromonas.*

## Résultats

Parmi les 81 patients inclus, 76 avaient une infection à *Aeromonas hydrophila* (94 %). Un pic d'incidence a été observé pendant la saison des pluies. Les patients étaient principalement des hommes (79, 64 %) avec un âge médian de 40 ans, non immunodéprimés. Une exposition à l'eau a été constatée dans 8 cas (10 %). Les morsures de serpent (16, 20 %) et les traumatismes routiers (22, 27 %) étaient les portes d'entrée les plus fréquentes. Dans l'analyse multivariée, les brûlures infectées ont été associées à un sur-risque d’évolution défavorable. Concernant le profil de résistance aux antibiotiques, 72 % des souches étaient résistantes à la ticarcilline. Seulement 2,6 % des souches étaient résistantes aux fluoroquinolones et aucune aux céphalosporines de troisième génération.

## Discussion et Conclusion

Dans l'environnement équatorial guyanais, les *Aeromonas* sont responsables d'infections cutanées, particulièrement chez des patients jeunes et immunocompétents. Diverses expositions telluriques et aquatiques peuvent être incriminées. Les fluoroquinolones ou les céphalosporines de troisième génération doivent être envisagées après une morsure de serpent, un contact tellurique ou toute infection cutanée dont l’évolution est défavorable après un traitement antibiotique de première ligne.

# Research article: JDI-02: Étude rétrospective de la fréquence des infections sexuellement transmissibles à Chlamydia et Gonocoque chez les patientes du Centre de santé de Maripasoula en 2019 et recherche de facteurs associés

**Published:** 2021-08-12

**Authors:** C. Etcheverry, A. Carbunar, D. Blanchet, M. Gaillet, N. Thomas, T. Boudon, V. Kiewsky, C. Michaud

**Affiliations:** 1 Service des CDPS - Centre hospitalier Andrée Rosemon, Cayenne, Guyane française, France; 2 Institut de santé publique, d’épidémiologie et de développement, Université de Bordeaux, Bordeaux, France; 3 Laboratoire de microbiologie, Centre hospitalier Andrée Rosemon, Cayenne, Guyane française, France

## Contexte et introduction

Les connaissances relatives aux infections sexuellement transmissibles (IST) par *C. trachomatis* et *N. gonorrhoeae* et leur surveillance épidémiologique sont des enjeux majeurs en santé sexuelle. La Guyane ne bénéficie pas d'un réseau de surveillance dédié. L'objectif principal de cette étude était d'estimer la fréquence des infections à *C. trachomatis* et *N. gonorrhoeae* chez les femmes consultant au centre de santé de Maripasoula en 2019. L'objectif secondaire consistait à rechercher les facteurs associés à la survenue de ces infections.

## Méthodologie.

Les données ont été recueillies rétrospectivement à partir des dossiers médicaux du centre de santé de Maripasoula, chez les femmes ayant consulté dans cette structure et bénéficié d'un test re-cherchant une infection à *C. trachomatis* et/ou *N. gonorrhoeae* en 2019. La population d’étude et la fréquence des infections ont été décrites. Les facteurs associés aux infections par *C. trachomatis* et *N. gonorrhoeae* ont été analysés selon le statut infectieux par une analyse statistique bivariée.

## Principaux résultats

Au total, 256 femmes ont été incluses. Près de 15 % des femmes présentaient une IST à *C. trachomatis* et/ou *N. gonorrhoeae* et la fréquence était plus élevée chez les femmes enceintes (19,6 %). La fréquence des infections à *C. trachomatis* était de 10,9 % (IC95 %: 7,4–15,4) et celle des infections à *N. gonorrhoeae* de 4,7 % (IC95 %: 2,5–8,1). Les principaux facteurs associés à la survenue de ces infections étaient l’âge inférieur à 25 ans (p<0,01), le type de couverture sociale (p=0,02) et l'absence de travail dans l'orpaillage (p=0,02). Les femmes nées en Guyane étaient sur-représentées par rapport aux femmes brésiliennes dans la population des sujets infectés.

## Discussion et Conclusion

Les IST à *C. trachomatis* et *N. gonorrhoeae* au sein de la population féminine du centre de santé de Maripasoula apparaissent comme un problème de santé publique par leurs fréquences, leurs enjeux de dépistage et de prise en charge. Ce travail peut constituer une première étape dans l'amélioration des connaissances relatives à ces infections dans la région. L’évaluation de leur prévalence nécessiterait néanmoins d’être approfondie et étendue à la population masculine.

# Research article: JDI-16: Le risque d'accidents vasculaires cérébraux chez les enfants et adolescents atteints d'un syndrome drépanocytaire majeur en Guyane Française

**Published:** 2021-08-12

**Authors:** J. Gargot, M.-C. Parriault, B. Oubda, J. Clouzeau, N. Elenga

**Affiliations:** 1 Service de pédiatrie générale– Centre hospitalier de Cayenne Andrée Rosemon, Cayenne, Guyane française, France; 2 Centre d'investigation clinique Antilles Guyane, CIC INSERM 1424– Centre hospitalier de Cayenne Andrée Rosemon, Cayenne, Guyane française, France; 3 Service de pédiatrie générale– Centre hospitalier de Kourou, Kourou; 4 Service de pédiatrie générale– Centre hospitalier de l'Ouest Guyanais, Saint Laurent du Maroni

## Introduction

En Guyane, un nouveau-né sur 227 serait atteint d'un syndrome drépanocytaire majeur (SDM): c'est l'incidence à la naissance la plus élevée de France. Bien que les accidents vasculaires cérébraux (AVC) soient une des complications de la drépanocytose avec une morbi-mortalité importante, ils ont été peu décrits en Guyane. Dans la littérature, le risque d'AVC est estimé à 11 % avant 20 ans chez les SS/Sβ0-thalassémie. Un dépistage cérébro-vasculaire par doppler trans-crânien (DTC) permet de prévenir efficacement la survenue d'AVC. Il a été mis en place depuis 2009 en Guyane chez les enfants drépanocytaires à risque (SS/Sβ0-thalassémie) âgés de 12-18 mois à 16 ans L'objectif de cette étude était d'estimer le risque d'AVC chez les enfants atteints d'un SDM en Guyane.

## Matériel et Méthode

Notre étude épidémiologique rétrospective incluait les enfants avec SDM nés en Guyane entre le 01/01/1992 et le 31/12/2002, non-opposés à l’étude, ayant un dossier médical dans l'un des trois hôpitaux publics de Guyane.

## Résultats

Cent-vingt-deux patients ont été inclus sur 218 dépistés. Il y avait 58 % de patients SS/Sβ0, 33 % de SC et 9 % de Sβ+. Les facteurs de risque d'AVC, tels l'absence d'alpha-thalassémie, l'hypertension artérielle, l'asthme et l'hypertrophie des végétations et amygdales, n’étaient pas significativement différents selon les génotypes. Il y avait 3 AVC ischémiques à un âge moyen de 6,9 ans et un AVC hémorragique à 9,2 ans. L'incidence cumulée d'AVC ischémique chez les enfants SS/Sβ0 était de 3,1 nouveaux cas pour 1 000 personnes-années, et le risque de survenue d'AVC clinique était de 6,4 % avant 15 ans et 3 mois.

## Conclusion

L'incidence et le risque d'AVC clinique retrouvés chez les enfants SS/Sβ0 étaient moins élevés que dans la littérature. Cela semble s'expliquer par une combinaison de différents facteurs, dont, en première ligne, la mise en place du dépistage par DTC.

# Research article: JDI-04: Attaques d'essaims d’« abeilles tueuses » en Guyane Française: l'importance d'une prise en charge rapide

**Published:** 2021-08-12

**Authors:** S. Geoffroy, A. Fremery, Y. Lambert, C. Marty, A. Nathalie

**Affiliations:** 1 Service des urgences, Cayenne Hospital, Cayenne, Guyane française, France; 2 Département universitaire de médecine générale, Université des Antilles, Pointe-à-Pitre, Guadeloupe, France; 3 Centre d'investigation clinique Antilles-Guyane (Inserm 1424), Hôpital de Cayenne, Cayenne, Guyane française, France; 4 Croix-Rouge Française, Cayenne, Guyane française, France,; 5 Centre Médical Inerarmées, Cayenne, Guyane française, France

En Guyane française, région française d'outre-mer située en partie en Amazonie, des abeilles « africanisées », espèce hybride d'abeilles brésiliennes connue sous le nom d’« abeilles tueuses », sont observées depuis 1975. Depuis cette date, plusieurs cas nécessitant de longues durées d'hospitalisation ont été décrits, permettant de mieux comprendre les mécanismes physiopathologiques de cette envenimation particulière. Nous rapportons ici une série de 10 cas de patients attaqués simultanément par des centaines d'abeilles tueuses et immédiatement traités par une équipe médicale pré-hospitalière déjà sur place. Entre 75 et 650 dards ont été retirés par victime. Le traitement de référence de l'anaphylaxie par injection intramusculaire d'adrénaline, remplissage vasculaire et oxygénothérapie a été administré à tous les patients sans délai. Une description clinique a été fournie et des tests biologiques ont été réalisés immédiatement après l'envenimation. Nous constatons donc l'existence d'une réaction en deux phases, de type anaphylactique, bien contrôlée médicalement, suivie d'une réaction toxique systémique. Ainsi, tous nos patients ont quitté l'hôpital après 44 heures de surveillance sans complications ni séquelles. Enfin, nos données cliniques et biologiques suggèrent qu'une injection précoce d'adrénaline associée à un remplissage vasculaire a permis d’éviter une réaction en chaîne d'insuffisance rénale aiguë fonctionnelle, d'accumulation de myoglobine, de nécrose tubulaire aiguë et d'insuffisance rénale aiguë organique. Cette observation est renforcée par le prompt rétablissement et l'absence de complications ou de décès dans notre groupe de patients, malgré des niveaux d'envenimation décrits comme potentiellement mortels ailleurs dans la littérature. Ce cas démontre l'importance d'un traitement d'urgence par injection intramusculaire d’épinéphrine, disponible sous forme de stylo auto-administré à usage unique pour une utilisation en cas d'attaque d'essaim d'abeilles tueuses.

# Research article: JDI-07: Investigation d'une épidémie de leishmaniose cutanée chez des militaires en Guyane

**Published:** 2021-08-12

**Authors:** K. Henry, A. Mayet, M.A. Hernandez, M. Demar, R. Blaizot

**Affiliations:** 1 Laboratoire hospitalo-universitaire de parasitologie-mycologie, Centre hospitalier de Cayenne, Cayenne, Guyane française, France; 2 Service de Santé des Armées, DIASS de Guyane, Cayenne, Guyane française, France; 3 Centre national de référence des leishmanies, Laboratoire associé, Centre hospitalier de Cayenne, Cayenne, Guyane française, France; 4 Tropical Biome and Immunophysiopathology (TBIP), Université de Lille, CNRS, Inserm, Institut Pasteur de Lille, U1019-UMR9017-CIIL-Centre d'infection et d'immunité de Lille, Centre hospitalier de Cayenne, Cayenne, Guyane française, France; 5 Service de dermatologie, Centre hospitalier de Cayenne, Cayenne, Guyane française, France

## Introduction

La leishmaniose cutanée (LC) est endémique en Guyane, avec 200 à 300 cas/an, sporadiques, mais concentrés sur la saison des pluies. Les épidémies sont rares et peu investiguées. Un signal épidémique était émis le 15 mai 2020 avec 15 cas suspects du 3^e^ Régiment étranger d'infanterie (REI) et 9^e^ Régiment d'infanterie marine (RIMA) et ayant participé à un stage d'entraînement en forêt équatoriale (CEFE). Une investigation épidémique était réalisée.

## Méthodologie

Étaient définis comme cas les militaires participant au CEFE entre le 01/01/2020 et le 30/06/2020, avec lésions compatibles et au moins un prélèvement positif/évolution favorable sous traitement antileishmanien. Une investigation sur site était réalisée, ainsi qu'une description clinique et une phylogénie du gène Hsp70. Une étude cas-témoin comparait les facteurs de risques individuels avec des stagiaires du CEFE sur la même période, non contaminés.

## Résultats

Trente cas étaient confirmés. *Leishmania guyanensis* était la seule espèce retrouvée. La présentation la plus fréquente était ulcéreuse (90 %), au niveau des membres supérieurs (63 %). Huit cas (26 %) présentaient une mauvaise évolution sous Pentacarinat et nécessitaient une 2^e^ ligne par Ambisome, dont trois nécessitaient une 3^e^ ligne par Ambisome + Miltefosine. Deux sites au sein du CEFE étaient à l'origine des contaminations, près d'une activité de déforestation illégale. Les souches isolées ne constituaient pas un cluster à part. Vingt-deux cas et vingt-deux témoins répondaient au questionnaire. La participation durant la semaine 13 de 2020 était associée à l'infection (p> 0,001), mais aucun facteur comportemental. Le taux d'attaque du RIMA (28,9 %) était plus élevé que celui du REI (3,3 %), comme celui des stagiaires du CEFE (8 %) par rapport aux autres militaires de Guyane sur la même période (0,08 %).

## Discussion et Conclusion

L'absence de cluster génétique suggère une infection par de nombreux phlébotomes sur les sites déforestés. L'expérience des légionnaires pouvait constituer un facteur protecteur. Les évolutions cliniques défavorables laissent craindre l’émergence de souches résistantes aux traitements.

# Research article: JDI-09: Suivi pédiatrique des enfants des communes « de l'intérieur » de la Guyane: description de 9 ans de consultations

**Published:** 2021-08-12

**Authors:** L. Jouan, É. Martin, M. Nacher, V. Servas, F. Djossou, N. Elenga, F. Henaff

**Affiliations:** 1 Service de pédiatrie, Centre hospitalier de Cayenne Andrée Rosemon, Cayenne, Guyane française, France; 2 Service des CDPS – Centre hospitalier de Cayenne Andrée Rosemon, Cayenne, Guyane française, France; 3 Unité de maladies infectieuses et tropicales, Centre hospitalier Andrée Rosemon, Cayenne, Guyane française, France

## Introduction

La Guyane est un département français des Amériques. Elle a la particularité d'avoir des communes dites « de l'intérieur » isolées, accessibles seulement par voie aérienne ou fluviale. Les populations qui y résident sont majoritairement d'origine amérindienne ou noire marronne. Dans ces communes, l'offre de soins est desservie par les Centres délocalisés de prévention et de soins (CDPS) et certains spécialistes, dont des pédiatres, vont régulièrement y mener des consultations. L'objectif principal de notre étude était de faire une description épidémiologique des pathologies infantiles ayant nécessité un suivi ou au moins une consultation par les pédiatres en CDPS entre 2011 et 2019.

## Matériel et Méthode

Nous avons réalisé une étude épidémiologique descriptive, rétrospective, multicentrique, des consultations par les pédiatres dans les centres de santé en Guyane entre le 1^er^ janvier 2011 et le 31 décembre 2019.

## Résultats

Au total, 1 779 patients ont été analysés. En 2011, au début des missions de pédiatrie, 91 patients ont eu au moins une consultation avec un pédiatre alors que 319 patients étaient suivis par un pédiatre dans les CDPS en 2019. 62 % des consultations de pédiatrie sont des consultations ponctuelles. 23 décès ont été recensés entre 2011 et 2019; cela représente 1,4 % des enfants suivis. 164 patients ont été perdus de vue. Les pathologies les plus fréquemment retrouvées sont les motifs cardiologiques, infectiologiques, les retards de développement, la prématurité et les pathologies génétiques, dont la drépanocytose.

## Discussion

Cette étude montre que le taux d'enfants suivis par les pédiatres en mission dans les CDPS est important: grâce aux données de l'INSEE de 2017, nous estimons que 7,1 % de la population 0-14 ans de Maripasoula est suivie pour une pathologie chronique. Les pathologies génétiques ou congénitales sont le 5^e^ motif de consultation spécialisée en pédiatrie dans les CDPS. Nous avons montré qu'il y a significativement plus de risque d’être atteint de syndrome drépanocytaire majeur sur le Maroni que sur l'Oyapock (p=0,0001). Ceci est complètement inhérent à l'origine ethnique des populations, plutôt noire-marronne sur le Maroni et amérindienne sur l'Oyapock, et aux connaissances sur les gènes des syndromes drépanocytaires majeurs. La prise en charge socio-médicale des patients trisomiques 21, semble nettement inférieure à celle attendue en France. Concernant les retards staturo-pondéraux et de langage, ils sont significativement plus nombreux à Camopi avec respectivement p=0,0001 et p=0,008. Le saturnisme pourrait être un facteur expliquant cette différence. Les cardiopathies congénitales sont également fréquentes en Guyane. Nous avons pu montrer grâce à ces données l'importance d'un suivi spécialisé dans les communes de l'intérieur de la Guyane.

# Research article: JDI-08: Sacro-illite septique à *Erysipelothrix rhusiopathiae* révélant une endocardite tricuspide. Cas clinique et revue de la littérature

**Published:** 2021-08-12

**Authors:** M. Kheng, J. F. Alexis, G. Walter, É. Mosnier, T. Malmontet, B. Moreau, Vilyn Tauch, G. Vesin, P. Abboud, M. Nacher, F. Roque, F. Djossou, L. Epelboin

**Affiliations:** 1 Unité de maladies infectieuses et tropicales, Centre hospitalier Andrée Rosemon, Cayenne, Guyane française, France, Guyane, France; 2 Service de cardiologie, Centre hospitalier Andrée Rosemon, Cayenne, Guyane française, France; 3 Ecosystèmes Amazoniens et Pathologie Tropicale, EA3593, Université de Guyane, Cayenne, Guyane française, France; 4 Aix Marseille Université, INSERM, IRD, SESSTIM, Sciences économiques et sociales de la santé et traitement de l'information médicale; Traitement de l'Information Médicale, Marseille, France; 5 Laboratoire de microbiologie, Centre hospitalier Andrée Rosemon, Cayenne, Guyane française, France; 6 Service de radiologie, Centre hospitalier Andrée Rosemon, Cayenne, Guyane française, France; 7 CIC INSERM 1424, Centre hospitalier Andrée Rosemon, Cayenne, Guyane française, France; 8 Service de chirurgie thoracique, Centre hospitalier Universitaire de Martinique, Fort de France, Martinique, France

## Introduction

Le réservoir de la bactérie *Erysipelothrix rhusiopathiae* est animal: porcs, bovins, poissons, crustacés et volailles sont ses principaux hôtes. Elle peut persister jusqu’à plusieurs mois dans leur environnement. L'Homme se contamine par contact avec les animaux. La plupart des cas sont liés à une exposition professionnelle. Trois entités cliniques ont été décrites chez l'Homme: une forme cutanée localisée, une forme cutanée diffuse et une forme systémique, avec bactériémie et/ou infection de sites habituellement stériles. En cas de bactériémie, une endocardite infectieuse est fréquemment constatée. L'arthrite septique et l'ostéomyélite ont été rarement décrites. Nous présentons ici un cas d'endocardite infectieuse à *E. rhusiopathiae,* révélé par une sacro-illite aiguë droite. Nous avons également passé en revue tous les cas d'infections ostéo-articulaires causées par *E. rhusiopathiae.*

## Cas clinique

Une femme de 53 ans, femme au foyer, sans antécédents médicaux, a été admise à l'hôpital de Cayenne, en raison d'une douleur à la hanche et à la cuisse droite et de fièvre. Une coxarthrose bilatérale et une sacro-illite droite étaient visibles sur le CT-scan, confirmées par l'IRM. Les hémocultures étaient positives pour *E. rhusiopathiae.* L’échocardiographie trans-oesophagienne a révélé une végétation tricuspide mobile. Le scanner thoracique a révélé des opacités alvéolaires pulmonaires bilatérales, compatibles avec des embolies septiques. Le diagnostic d'endocardite tricuspide infectieuse due à *E. rhusiopathiae* compliquée d'une embolie septique pulmonaire, révélée par une sacro-illite droite, a donc été retenu. La patiente a déclaré s’être coupé le majeur droit en coupant du poisson 2 mois auparavant. La Ceftriaxone a été introduite, remplacée par la ciprofloxacine après 3 semaines. L'antibiothérapie a duré 6 semaines. Aucune rechute ou complication n'a été identifiée au cours des 3 années suivantes.

## Revue de la littérature

Dix-huit cas d'infections ostéo-articulaires à *E. rhusiopathia*e ont été décrits dans la littérature, incluant arthrite, ostéomyélite, infections de prothèses. Aucun cas de sacro-illite n'a été décrit auparavant. La porte d'entrée a été identifiée dans 12 cas. La période d'incubation variait de quelques semaines à plusieurs années. Dans 9 cas, une histoire médicale spécifique (immunosuppression, alcoolisme chronique, diabète) a été retrouvée, et dans 4 cas, l'infection a eu lieu en présence de matériel orthopédique. Une bactériémie a été associée dans 3 cas et une endocardite infectieuse dans 2 cas.

Nous rapportons ici un cas atypique de sacro-illite aiguë révélant une endocardite tricuspide chez une femme sans antécédent médical ni matériel orthopédique, avec une exposition au pathogène deux mois auparavant.

# Research article: JDI-26: Maladie de Kaposi en Guyane Française: évolution épidémiologique et thérapeutique

**Published:** 2021-08-12

**Authors:** C. Lamoureux, K. Drak Alsibai, D. Sainte-Marie, R. Pradinaud, P. Couppié, R. Blaizot

**Affiliations:** 1 1. Service de dermatologie, C.H. Andrée Rosemon, Cayenne, Guyane française, France.; 2 2. Laboratoire d'anatomo-pathologie, CH Andrée Rosemon, Cayenne, Guyane française, France; 3 3. Tropical Biome and Immunophysiopathology (TBIP), U1019-UMR9017-CIIL, Cayenne, Guyane française, France

## Introduction

Les données sur la maladie de Kaposi (MK) en Guyane française (GF) sont rares et anciennes. Ce territoire présente des caractéristiques épidémiologiques uniques en Amérique du Sud en raison de son système de santé universel et de la présence de populations créoles, africaines, européennes et indigènes. L'influence des ethnies mixtes et des pandémies de VIH dans la prise en charge de la MK n'a pas été décrite sur ce territoire. Nos objectifs étaient d'analyser les caractéristiques cliniques, les résultats et l'incidence des différentes formes de MK en GF depuis 1969.

## Matériel et Méthode

Tous les patients vus à l'hôpital de Cayenne entre 1969 et 2019 avec un diagnostic de MK.

## Diagnostic

Clinique compatible +histologie compatible +/- immunohistochimie ou PCR HHV8 positive sur biopsie.

## Résultats

Cinquante-deux patients étaient inclus, soit 43 hommes et 9 femmes. Pic d'incidence de Kaposi épidémique dans les années 1990, puis diminution et stabilisation depuis les années 2000. Pic d'incidence de Kaposi endémique dans les années 1980 puis nette diminution depuis les années 2000 La forme épidémique était associée à: population HSH (p 0,0054), nationalité haïtienne (p=0,035). La forme endémique était associée à: présence de nodules cutanés/tumeurs (p 0,0001) lésions limitées au membre inférieur (p 65 ans (p 0,0001). Le taux de guérison complète était significativement plus élevé (p = 0,02) dans la MK endémique (70,6 %) vs épidémique (22,7 %). Un patient amérindien présentait une transformation maligne (anaplasique) de sa MK initiale non classée.

## Discussion

Nous avons observé une diminution de l'incidence de la MK épidémique à partir des années 2000. Elle demeure néanmoins la principale forme de MK en Guyane est actuellement liée au VIH. La MK endémique est en voie de disparition. Il s'agit de la première description d'une transformation anaplasique en Amérique du Sud, peut-être liée à un facteur génétique chez un patient amérindien est actuellement liée au VIH. Il s'agit également de la première étude qui décrit la variante anaplasique KS chez un Amérindien.

# Research article: JDI-06: Facteurs associés à la non-récupération des résultats de dépistage des IST au CeGIDD de Saint-Laurent-du-Maroni (Guyane) après une première consultation entre le 1^er^ Janvier 2017 et le 3 Octobre 2019

**Published:** 2021-08-12

**Authors:** S. Lemonnier, M. Mezzarobba, J. Robert, C. Agostini

**Affiliations:** 1 CeGIDD du Centre hospitalier de l'Ouest Guyanais, Saint-Laurent du Maroni; 2 Département universitaire de médecine générale, Université des Antilles, Pointe-à-Pitre, Guadeloupe, France; 3 Economiste de la santé/statisticien à Cemka, aide à titre personnel; 4 Biostatisticienne, Centre hospitalier Universitaire de Nîmes, Nîmes, aide à titre personnel; 5 Département de Santé Publique, Centre hospitalier de l'Ouest Guyanais, Saint-Laurent du Maroni

## Introduction

La Guyane reste confrontée à des difficultés sanitaires d'une ampleur particulière. Son enclavement, l'importance de la natalité et de l'immigration, l'exposition d'une part importante de la population à une forte vulnérabilité socio-économique, les atteintes avérées aux droits sexuels et la persistance de croyances erronées, facteurs de discriminations et de stigmatisation, sont responsables d'une forte prévalence du VIH et des autres IST. Ces facteurs sont amplifiés dans l'Ouest Guyanais par sa position particulière frontalière avec le Suriname. La persistance d'obstacles à l'offre de prévention et de dépistage de ces pathologies limite l'entrée dans le système de soins pour les jeunes, les femmes et les migrants. Notre étude porte sur le CeGIDD (Centre gratuit d'information, de dépistage et de diagnostic) du Centre hospitalier de l'Ouest Guyanais à Saint Laurent du Maroni. Conforme à la difficulté d'intégrer certaines populations dans le système de soins, le taux de perdus de vue du centre est de 30 %. Les objectifs de cette étude étaient d'une part d'analyser le profil des personnes qui ne retournent pas en consultation de rendu des résultats à sept jours (objectif principal de l’étude) puis trente jours après leur dépistage, de déterminer leur taux de positivité, et de relever auprès d'elles quelques facteurs limitant leur retour en consultation.

## Méthodologie

Nous avons réalisé une étude rétrospective, monocentrique et observationnelle incluant toutes les personnes ayant effectué un dépistage entre le 1^er^ janvier 2017 et le 3 octobre 2019 au CeGIDD en recueillant des variables en lien avec des aspects sociaux, démographiques et de pratiques sexuelles ainsi que les résultats des tests effectués. Dans le cadre du dernier objectif secondaire un recueil prospectif par questionnaire a été proposé systématiquement à tous les consultants du 02/03/2020 au 16/03/2020.

## Résultats

Sur la période d’étude, 1 818 personnes ont consulté. Concernant l'objectif principal, les facteurs liés à la non-récupération des résultats sept jours après le dépistage étaient être une femme, être sans profession, consulter pour un test de grossesse ou un test de routine, avoir une exposition au risque de plus de trois mois, être monogame, avoir des tatouages/piercings. À l'inverse avoir eu un rapport non protégé, un incident de préservatif ou un AEV ainsi qu'une utilisation variable du préservatif pour la pénétration anale étaient liés positivement au fait de revenir. Concernant les objectifs secondaires, les facteurs liés à la non-récupération des résultats à trente jours étaient d’être d'origine française (hors métropole), ne pas avoir déclaré de téléphone, être en couple, avoir une utilisation variable du préservatif et déclarer le multipartenariat avec des femmes. Consulter pour un rapport non protégé ou à la demande du partenaire étaient liés positivement au fait de revenir chercher ses résultats. À 7 jours, les consultants positifs au gonocoque revenaient moins, à trente jours cela concernait les consultants positifs à l'hépatite B. Enfin, concernant les facteurs pouvant limiter le retour en consultation, tous les répondants souhaitaient obtenir leurs résultats. La mise en avant de quelques freins ainsi que le souhait du SMS comme outil de rappel a ouvert la voie à des explorations ultérieures.

## Conclusion

Cette première étude sur les perdus de vue au CeGIDD du Centre hospitalier de l'Ouest Guyanais a permis de mettre en avant plusieurs facteurs liés à la non-récupération, permettant ainsi de cibler les consultants à risque, ainsi que de valider l'utilisation du téléphone comme outil de rappel, ouvrant la discussion sur les autres moyens de rappel et de rendu des résultats des tests. Cette étude permettrait la réalisation d'autres ultérieures sur les causes de perdus de vue et les outils de rappel/rendu de résultats applicables dans la région.

# Research article: JDI-18: Évaluation du SD Malaria Ag P.f/Pan^®^ comme test de diagnostic rapide (TDR) du paludisme en Amazonie Française

**Published:** 2021-08-12

**Authors:** S. Lemonnier, A. Fremery, H. Kallel, J.-M. Pujo

**Affiliations:** 1 Service d'accueil des urgences, SAMU-SMUR 973, Centre hospitalier de Cayenne, Cayenne, Guyane française, France; 2 Service de réanimation polyvalente, Centre hospitalier de Cayenne, Cayenne, Guyane française, France

## Introduction

L'OMS a classé le paludisme comme maladie transmissible parmi les priorités en matière de santé publique mondiale. Dans le monde entier, il est constaté une augmentation de la délétion du gène PfHRP2. Cette mutation impacte la performance des tests rapides à diagnostiquer les patients infectés par *P. falciparum* et elle est responsable de faux négatifs. L'objectif de cette étude est d’évaluer la pertinence diagnostique des tests de diagnostic rapide (TDR) détectant la présence des antigènes pan-pLDH et PfHRP2 utilisés dans le diagnostic du paludisme dans les centres de notre région.

## Matériel et Méthode

Nous avons effectué une analyse rétrospective de janvier 2016 à décembre 2019 dans notre laboratoire de microbiologie comprenant tous les TDR ainsi que les analyses de sang sur lame (BFT) basé sur un examen microscopique du sang prélevé pour le diagnostic du paludisme.

## Résultats

Durant cette période, 12 984 échantillons sanguins pour le diagnostic du paludisme ont été prélevés. Le TDR et le BFT ont été effectués dans 10 873 cas et se sont révélés positifs respectivement dans 773 cas (7,1 %) contre 791 cas (7,3 %). *P. falciparum* a été identifié dans 125 cas (16,1 %) avec le TDR contre 105 (13,3 %) au BFT et *P. vivax* dans 648 cas (83,9 %) contre 673 cas (85,1 %). L’évaluation globale de la précision du diagnostic du TDR dans le diagnostic du paludisme montre une VPP et une VPN supérieures à 95 %, sauf pour la VPP du TDR dans le diagnostic du paludisme à *P. falciparum* (88 %). Dans l'ensemble, le taux de concordance entre le TDR et le BFT était de 99,5 %.

## Discussion et Conclusion

Notre étude visait à évaluer le test SD Malaria Ag P.f/Pan^®^ qui est le TDR actuellement utilisé dans les Centres délocalisés de prévention et de soins de notre région. Contrairement aux pays frontaliers, nous ne sommes pas encore concernés par la délétion du gène FpHRP2. Par ailleurs, en 2020, dans son Bulletin épidémiologique périodique, l'Organisation de santé publique « Santé Public France », a signalé une augmentation significative des cas de paludisme importés des pays voisins et du continent africain. Ce phénomène pourrait engendrer une résurgence du *Plasmodium falciparum* sur le territoire.

# Research article: JDI-12: L'infection par le VIH dans l'Ouest Guyanais: étude comparative du statut immunologique au diagnostic entre les patients dépistés au Centre hospitalier d'André Rosemon à Cayenne (CHAR) et ceux dépistés au Centre hospitalier de l'Ouest Guyanais à Saint-Laurent-du-Maroni (CHOG) ente 2007 et 2017

**Published:** 2021-08-12

**Authors:** E. Louaisil, M. Nacher, J. Adoissi, F. Samou Fantcho, O. Epaulard, Y. Gaboreau, É. Ouedraogo

**Affiliations:** 1 Service de médecine polyvalente, maladies infectieuses et tropicales, Centre hospitalier de l'Ouest Guyanais, Saint-Laurent-du-Maroni, Guyane française, France; 2 Département universitaire de médecine générale, Université Grenoble Alpes, Grenoble, France; 3 Centre d'investigation clinique Antilles-Guyane, Guyane française, France; 4 COREVIH Guyane, Centre hospitalier André Rosemon, Cayenne, Guyane française, France; 5 Service de maladies infectieuses et tropicales, Centre hospitalier Universitaire Grenoble Alpes, Grenoble, France; 6 Fédération d'infectiologie multidisciplinaire de l'Arc Alpin, Université Grenoble Alpes, Grenoble, France; 7 Département de médecine générale, Université Grenoble Alpes, Grenoble, France; 8 Service de Maladies infectieuses et tropicales, Hôpital Avicenne, Bobigny, France

## Introduction

La Guyane, département où l'incidence du VIH est la plus importante de France, déclare 900 cas/millions d'habitants. L'objectif de cette étude était de comparer le statut immunologique des patients séropositifs pour le VIH au diagnostic, entre les sujets dépistés au CHAR (Centre hospitalier André Rosemon, Cayenne) et ceux dépistés au CHOG (Centre hospitalier de l'Ouest Guyanais) de 2007 à 2017. L'objectif secondaire était d'identifier les facteurs de risques de dépistage tardif.

## Matériel et Méthode

Nous avons mené une étude observationnelle rétrospective, bicentrique. Le critère de jugement principal était le statut immunologique au diagnostic, critère composite associant le stade CDC et le taux de lymphocytes T CD4. Le dépistage était tardif si, au diagnostic, les CD4 étaient inférieurs à 200/mm^3^ et/ou si le stade CDC était C.

## Résultats

Les patients du bras CHOG étaient diagnostiqués plus tardivement que ceux du CHAR (p<0,05 OR 2,06 IC [1,52-2,80]). Les facteurs de risques de dépistage tardif au CHOG étaient le sexe masculin (OR 3,27 IC [2,01-5,31]), un âge entre 30 et 49 ans (OR 2,54 IC [1,58-4,08]), une co-infection au VHB et/ou VHC (OR 3,40 IC [1,07-10,80]). La grossesse était un facteur protecteur (OR 0,14 IC [0,06-0,33]). Dans le groupe CHAR, être né au Surinam était un facteur de risque de dépistage tardif (OR 2,88 IC [1,11-7,46]).

## Discussion

L'infection par le VIH est diagnostiquée à un stade plus tardif au CHOG qu'au CHAR. Au Suriname la thérapie antirétrovirale n'a été recommandée systématiquement qu’à partir de 2015. La frontière poreuse séparant l'Ouest Guyanais du Suriname, apparaît comme un lieu de transmission active.

## Conclusion

Une politique de dépistage systématique et précoce, ciblant les hommes nés au Suriname, au même titre que les femmes au début de leur grossesse doit être instaurée pour faire face à une épidémie encore active.

# Research article: JDI-22: La lèpre comme syndrome inflammatoire de reconstitution immune chez les patients infectés par le VIH sous traitement antirétroviral en Guyane Française: étude rétrospective de 1999 à 2020

**Published:** 2021-08-12

**Authors:** A. Mouchard, P. Couppié, J. Graille, R. Blaizot, C. Bertin

**Affiliations:** 1 Service de dermatologie, Centre hospitalier Andrée Rosemon, Cayenne, Guyane française, France; 2 Tropical Biome and Immunophysiopathology (TBIP), Université de Guyane, Guyane française, France

## Introduction

La lèpre est une maladie ré-émergente en Guyane (incidence de 1 cas/10 000 habitants de 2007 à 2013), où l'infection par le VIH touche près de 1 % de la population. Chez les patients vivants avec le VIH (PvVIH), l'incidence de la lèpre est 18 fois plus importante dans les 3 mois suivants l'introduction des antirétroviraux (ARV) que chez les PvVIH non traités. La lèpre peut se présenter en tant que syndrome inflammatoire de reconstitution immune (IRIS). Cet IRIS est défini par l'apparition chez un patient infecté par le VIH profondément immunodéprimé (CD4 1 log et augmentation des CD4).

## Méthodologie

Étude rétrospective de 1999 à 2020 étudiant tous les patients VIH ayant développé une lèpre dans les 6 mois suivant la mise sous ARV. Le diagnostic reposait sur la clinique et l'examen anatomopathologique. Les caractéristiques cliniques, biologiques, histologiques et l'efficacité de la polychimiothérapie (PCT) ont été recueillies.

## Résultats

Six PvVIH masculins de 24 à 54 ans ont présenté une lèpre en médiane 8 semaines (1-24 semaines) après l'introduction des ARV. Les formes cliniques étaient borderline tuberculoïde (4/6), tuberculoïde (1/6), borderline borderline (1/6) selon la classification de Ridley et Jopling, et majoritairement multibacillaires (5/6) selon celle de l'OMS. Deux patients ont présenté un état réactionnel de type 1 au diagnostic, et 2 autres au cours de la PCT. Parmi ces 4 patients, 3 ont développé des ulcérations des lésions initiales et 2 ont nécessité une corticothérapie générale en raison d'une névrite associée. L’évolution a été favorable pour 5/6 patients (1 perdu de vue), en un délai médian de 15 mois, sans récidive.

## Discussion

Rare, la survenue d'une lèpre en tant qu'IRIS chez les PvVIH sous ARV doit être évoquée systématiquement y compris devant des lésions cutanées atypiques chez les patients originaires de zones d'endémie lépreuse. La présentation est souvent classique, majoritairement sous forme borderline tuberculoïde, fréquemment accompagnée ou rapidement suivie d'un état réactionnel de type 1, parfois intense avec ulcérations mais d’évolution favorable sous PCT de durée usuelle.

# Research article: JDI-25: Toxoplasmoses sévères chez l'immunocompétent et virulence des souches de *Toxoplasma gondii*: une revue mondiale

**Published:** 2021-08-12

**Authors:** G. Pasquier, D. Blanchet, M. Pierre-Demar

**Affiliations:** Laboratoire hospitalo-universitaire de parasitologie et mycologie, Centre hospitalier de Cayenne, Cayenne, Guyane française, France

## Introduction

Des infections sévères dues à *Toxoplasma gondii* sont fréquemment rapportées chez les patients immunodéprimés, mais restent rares chez les immunocompétents. Cependant, plusieurs séries de cas ont été décrites en Guyane française chez ces derniers et ont été associées à des souches atypiques hautement virulentes. Mais qu'en est-il ailleurs dans le monde ? Pour y répondre, nous avons réalisé une revue globale de la littérature visant à évaluer le lien entre la sévérité des toxoplasmoses chez l'immunocompétent et la virulence des souches.

## Méthodologie

Toutes les souches publiées et génotypées avec au moins 5 marqueurs de RFLP ou de microsatellites ont été retenues pour la revue, soit 4 297 souches tirées de 289 publications. La virulence de chaque génotype a été déterminée en fonction des données de la littérature lorsqu'elles étaient disponibles. Cent-cinquante-et-un de toxoplasmoses sévères chez l'immunocompétent issus de 113 articles ont été sélectionnés.

## Résultats

Les régions avec la plus grande proportion de souches virulentes se situent en zone tropicale: Amérique du Sud, Afrique de l'Ouest, Afrique centrale et Asie du Sud-Ouest. Néanmoins, la plupart des cas de toxoplasmoses sévères chez l'immunocompétents ont été décrits en Europe (57/151), en Amérique du Sud (52/151) et en Amérique du Nord (20/151). Les principales atteintes d'organes étaient pulmonaires (59/151), neurologiques (54/151) et cardiaques (51/151).

Au final, aucune corrélation n'a pu être établie entre l'origine géographique des cas et la présence de souches virulentes (p=0,60, ρ=0,20 coefficient de Pearson).

# Research article: JDI-13: Intérêt diagnostique de l'utilisation du β-D-glucane dans l'histoplasmose chez les patients VIH, en Guyane Française, étude rétrospective de 2016 à 2020

**Published:** 2021-08-12

**Authors:** A. Pignata, D. Blanchet, M. Demar, T. Bonifay, H. Kallel, P. Couppié, A. Adenis, F. Djossou, L. Epelboin

**Affiliations:** 1 Service de maladies infectieuses et tropicales, centre hospitalo-universitaire de Rouen, Rouen, France; 2 Service de maladies infectieuses et tropicales, Centre hospitalier intercommunal Elbeuf-Louviers, Rue du Docteur Villers 76410 Saint-Aubin-lès-Elbeuf, France; 3 Département de biologie, Centre hospitalier de Cayenne Andrée Rosemon, Cayenne, Guyane française, France; 4 Unité sanitaire en milieu pénitentiaire, Centre pénitentiaire de Guyane et Centre d'investigation clinique Antilles Guyane Inserm 1424, CH de Cayenne, Guyane; 5 Service de réanimation, Centre hospitalier de Cayenne Andrée Rosemon, Cayenne, Guyane française, France; 6 Service de dermatologie, Centre hospitalier de Cayenne Andrée Rosemon, Cayenne, Guyane française, France; 7 Service de maladies infectieuses et tropicales, Centre hospitalier de Cayenne Andrée Rosemon, Cayenne, Guyane française, France

## Introduction

L'histoplasmose, infection par *Histoplasma capsulatum* variant *capsulatum* (Hcc), endémique en Amérique latine, représente la première cause de stade sida et est la première cause de mortalité chez le patient VIH. Actuellement, peu d'outils diagnostiques sont disponibles dans cette pathologie fréquente et avec une lourde mortalité. L'objectif de cette étude est de déterminer les caractéristiques diagnostiques du β-D-glucane (BDG), dans le cadre du diagnostic d'histoplasmose chez le patient VIH.

## Matériel et Méthode

Centre quatre-vingt-onze patients vivants avec le VIH et ayant eu un prélèvement de BDG ont été recueillis rétrospectivement sur la période s’étendant du 1^er^ janvier 2016 au 31 janvier 2020, au Centre hospitalier de Cayenne.

## Résultats

Sur les 191 patients, 44 (23 %) avaient des BDG supérieurs à 80 mg/ml, 67 (35 %) supérieurs à 40 pg/ml et 33 (soit 17 %) avait une histoplasmose prouvée ou probable (HPP). Les BDG était significativement supérieurs dans le groupe avec histoplasmose que dans la population témoin (p<0,05). La sensibilité (Se), spécificité (Sp), valeur prédictive positive (VPP) et valeur prédictive négative (VPN) des BDG pour une norme à 40 pg/ml dans l'histoplasmose étaient, respectivement de 60, 70, 30 et 89 %, de 42, 81, 31 et 87 % pour une norme fixée à 80 pg/ml et de 50, 91, 75 et 77 % dans le cadre des infections fongiques invasives pour une norme fixée à 80 pg/ml. Les cytopénies, la dissociation ferritine/fibrinogène et la présence d'anomalies radiologiques étaient significativement associées à l'histoplasmose (p<0,05). L'association des BDG supérieurs à 40 pg/ml avec la présence d'organomégalie avaient une spécificité de 87 % et une VPN de 88 % pour l'histoplasmose prouvée ou probable.

## Conclusion

L'utilisation des BDG, avec une norme fixée à 40 pg/ml doit être envisagée dans le cadre du diagnostic d'histoplasmose, avec une Sp à 70 % et une VPN à 89 %. La Sp de ce dosage est de 87 % avec une VPN à 88 % en association avec la présence d'une organomégalie.

# Research article: JDI-19: Description épidémiologique et évaluation de la prise en charge des Body-Packers au Centre hospitalier de Cayenne de 2016 à 2019

**Published:** 2021-08-12

**Authors:** V. Piriou, C. Bonnefoy, K. Hamiche, K. Hatem, J. M. Pujo, A. Fremery

**Affiliations:** 1 Service d'accueil des urgences, Centre hospitalier de Cayenne, Cayenne, Guyane française, France; 2 Service de pédiatrie, Centre hospitalier de Cayenne, Cayenne, Guyane française, France; 3 Unité médico-JUDICIAIRE, Centre hospitalier de Cayenne, Cayenne, Guyane française, France; 4 Service de réanimation polyvalente, Centre hospitalier de Cayenne, Cayenne, Guyane française, France

## Introduction

Notre région constitue une plaque tournante du trafic international de stupéfiants. Le transport in corpore ou Body-packing est un des transports privilégiés par les narcotrafiquants. Le passage de cocaïne in corpore nécessite systématiquement une prise en charge hospitalière, mais les recommandations internationales à ce sujet sont pauvres et discordantes. Les objectifs de cette étude sont la description épidémiologique du Body-packing dans notre région et l’évaluation de sa prise en charge.

## Matériel et Méthode

Étude observationnelle rétrospective mono-centrique incluant les patients admis au Service d'accueil des urgences (SAU) et à l'Unité médico-judiciaire (UMJ) de notre Centre hospitalier (CH) du 1^er^ janvier 2016 au 31 décembre 2019 pour ingestion in corpore de boulettes de cocaïne. Les patients pour lesquels le transport in corpore de cocaïne a été infirmé ont été exclus.

## Résultats

Sur la période, 668 personnes ont été adressées au SAU et à l'UMJ par les services des douanes pour suspicion de body-packing. Parmi elles, 215 ont été exclues devant l'absence de boulettes à l'abdomen sans préparation (ASP) et 4 patients ont été exclus, car porteur de boulettes de cannabis. In fine, 449 patients ont été admis pour ingestion d'ovule de cocaïne soit une augmentation de 60 % par rapport aux années précédentes. L’âge moyen était de 25,2 ans avec un sex-ratio H/F de 2,21. Parmi eux, 13,7 % se sont compliqués sans mise en jeu du pronostic vital. Le nombre de boulettes moyen était similaire entre la population avec et sans complications (respectivement 54,1 [50,9-57,4] et 57,8 [48,9-66,6], p=0,22). La présence de cocaïne dans les urines n'a pas montré d'association significative avec la survenue complications (OR 0,5, IC95 % [0,1-1,8], p=0,238). En comparaison au scanner (gold standard), la sensibilité de l'ASP était de 44,1 %. La durée moyenne de séjour était significativement plus longue si l'ASP initial présentait 50 boulettes ou plus (p=0,0001).

## Conclusion

Cette étude a montré un faible taux de complications, dont une majeure partie seulement pour stagnation digestive et recours à l'endoscopie. Les durées moyennes de séjour sont relativement courtes, du fait du faible taux de complications et de l'absence de gravité de celles-ci. L'emballage de type 3 de qualité micro-industrielle, quasi exclusivement utilisé dans le body-packing dans notre région, semble être le principal facteur à cette réduction de complications.

# Research article: JDI-10: Évaluation de l'exposition au plomb chez les moins de 18 ans en Guyane de 2011 à 2018

**Published:** 2021-08-12

**Authors:** A. Truffault, T. Succo, C. Rousseau, A. Andrieu, A. Biola-Vidamment

**Affiliations:** 1 Cellule régionale de santé publique France en Guyane; 2 Université Paris Saclay, UFR de Pharmacie à Chatenay-Malabry; 3 Laboratoire de toxicologie, Université Paris Saclay, UFR de Pharmacie à Chatenay-Malabry

## Introduction

Depuis juin 2011 et la découverte d'un cas de saturnisme sévère chez une enfant de 3 ans, le saturnisme est considéré comme un problème de santé publique majeur en Guyane. L'objectif de ce travail est d'estimer le niveau d'intoxication au plomb chez les mineurs, de caractériser les populations touchées et de mettre en évidence les sources d'exposition au plomb chez les mineurs en Guyane.

## Méthodologie

Notre analyse de plombémies de 2011 à 2018 s'est basée sur la base de données du Système national de surveillance des plombémies de l'enfant (SNSPE), mais aussi sur la compilation des prélèvements réalisés pendant les enquêtes environnementales par l'ARS de Guyane autour des déclarations obligatoires.

## Résultats

Pour 3 827 plombémies, dont 3 184 primodépistages (83 %), nous avons identifié 949 cas incidents. La moyenne géométrique des plombémies est de 37,4 μg/l. Les régions de l'Oyapock et du Maroni semblent être plus touchées que le littoral. Contrairement aux facteurs de risque identifiés par l'analyse du SNSPE; la littérature, ainsi que les enquêtes environnementales, suggèrent une exposition multifactorielle à composante majoritaire alimentaire comme source d'intoxication principale au plomb en Guyane.

## Discussion et Conclusion

Le niveau d'imprégnation du plomb en Guyane est alarmant par rapport à celui mesuré en métropole. Les causes sont encore mal définies, mais on identifie des sources d'intoxication différentes de celles de la métropole. Les sources retrouvées ne suffisent que rarement à expliquer à elles-seules le niveau de plomb observé chez les enfants. La situation nécessiterait une poursuite des investigations en profondeur, dont une analyse des différentes sortes de manioc et des sols. Seules une coopération et une écoute des différentes communautés guyanaises concernées, ainsi qu'une sensibilisation des professionnels de santé, permettront d'apporter des solutions efficaces et pertinentes.
